# RECEIPT OF CHEMOTHERAPY AND COGNITIVE DECLINE AMONG MEDICARE BENEFICIARIES IN THE US HEALTH AND RETIREMENT STUDY

**DOI:** 10.1093/geroni/igad104.0089

**Published:** 2023-12-21

**Authors:** Ashly Westrick, Mohammed Kabeto, Lindsay Kobayashi

**Affiliations:** University of Michigan School of Public Health, Ann Arbor, Michigan, United States; University of Michigan, Ann Arbor, Michigan, United States; University of Michigan School of Public Health, Ann Arbor, Michigan, United States

## Abstract

Research on cancer and cognitive aging outcomes has been inconsistent, with clinic-based studies showing short-term cognitive declines after chemotherapy, and population-based epidemiological studies observing inverse associations between most cancers and subsequent cognitive aging. We aimed to determine the effect of chemotherapy on cognitive decline and mortality among Medicare beneficiaries in the population-based US Health and Retirement Study (HRS). Using data from HRS-Medicare linked participants ≥65, we identified 858 incident breast, prostate, and kidney cancer diagnoses from 2000 to 2016. Cognitive function was measured using the modified telephone interview for cognitive status (range 0 to 27). Cancer treatment (chemotherapy only, other treatments, and no treatment) was obtained from Medicare claims. We used joint modeling to simultaneously model the association between cancer treatment with cognition decline and mortality. Of 858 participants, 38.7% had breast cancer, 52% had prostate cancer, and 11% had kidney cancer. The mean age at diagnosis was 72.8 years (SD=7.62). 31.7% (n=272) received chemotherapy only, 39.5% (n=339) received other treatment, and 28.8% (n=247) received no treatment. In pooled analyses, receipt of chemotherapy (vs. no treatment) was not significantly associated with cognitive decline (β: 0.08; 95% CI: -0.59, 0.76) but was significantly associated with mortality (HR: 1.39; 95% CI: 1.04, 1.88). In joint modeling, the overall hazard ratio for the receipt of chemotherapy (vs. no treatment) was 1.38 (95% CI: 1.02, 1.88). We found that receipt of chemotherapy was associated with an increased hazard of mortality, but not cognitive decline, compared to those who received no treatment.

